# DVL regulation of tissue-specific aromatase transcripts in breast cancer

**DOI:** 10.18632/oncotarget.26496

**Published:** 2018-12-25

**Authors:** Heather M. O’Hagan

**Affiliations:** Heather M. O’Hagan: Medical Sciences Program, Indiana University School of Medicine, Bloomington, Indiana, USA

**Keywords:** aromatase, CYP19A1, DVL, breast cancer, estrogen

Breast cancer is the most common cancer in women in the United States. The majority of breast cancers are hormone receptor positive, with around 70% expressing the estrogen receptor (ER) and progesterone receptor [1]. Excessive estrogen exposure is a well-known risk factor for breast cancer and drives increased proliferation of ER+ tumors. Aromatase is a cytochrome P450 class monooxygenase enzyme that converts androgens to estrogens. The increased activity of aromatase enzymes has long been associated with tumorigenesis as well as other diseases caused by elevated estrogen levels. Aromatase activity has also been found in cancers not normally connected to hormone dependency. To target the reliance of tumors on estrogen, aromatase inhibitors such as anastrozole, letrozole, and exemestane are used to treat post-menopausal women with ER+ breast cancer [1]. Treatment with aromatase inhibitors results in reduced estrogen levels in blood and plasma and has significantly reduced recurrence of breast cancer in these patients. CYP19A1 is the gene that encodes the aromatase enzyme making understanding its regulation important to the study of cancer and other hormone-related diseases.

In a recent publication Castro-Piedras et al. set out to further understand the transcriptional regulation of CYP19A1 in breast cancer cells [2]. The 5’ UTR of CYP19A1 contains ten identified alternative untranslated first exons that are regulated by tissue-specific promoters for the placenta, adipose tissue, brain, skin, bone, and gonads [3]. Each tissue-specific isoform has identical coding regions but different 5’ untranslated first exons. The tissue-specific promoters are regulated by different transcription factors and affected by local hormones and signaling events. This differential regulation results in tissue-specific aromatase expression and estrogen biosynthesis. In breast cancer in postmenopausal women estrogen can be synthesized by the tumor tissue, surrounding stroma, or other body sites such as subcutaneous adipose tissue. Furthermore, in tumors CYP19A1 transcription loses tissue-specific regulation and can be driven by multiple promoters. The interaction between malignant epithelial cells and the cells in the microenvironment increases aromatase expression from multiple promoters resulting in high estrogen production in the area surrounding the tumor.

Sirtuin-1 (SIRT1) is a lysine deacetylase that has been implicated in aberrant gene silencing in cancer cells [4, 5]. In previous work the Pruitt group demonstrated that SIRT1 regulates CYP19A1 expression in breast cancer cells and that SIRT1, β-catenin and Dishevelled (DVL) coregulate Wnt signaling [6, 7]. Wnt pathway signaling is critical for development and tissue homeostasis but also becomes dysregulated in many different cancer types, including breast. DVL proteins are mediators of Wnt signaling that integrate cues from many Wnt ligands, receptors and co-receptors. The canonical role of DVL is in the cytoplasm where it promotes β-catenin stabilization. However, several recent studies have demonstrated that DVL can translocate to the nucleus, interact with c-Jun and β-catenin, and bind to the promoters of Wnt target genes [8].

This background knowledge led Castro-Piedras et al. to hypothesize that nuclear DVL regulates CYP19A1 expression in breast cancer cells. Using a panel of breast cancer cell lines that represent ER+ and ER- types they found that breast cancer cells express several different CYP19A1 transcripts, including ovary, adipose and placental transcripts [2]. Their study mainly focused on the less well-understood placental pI.1 transcript because it along with pII have been shown to corelate best with protein expression and therefore may have the most biologically relevant effect on tumor progression. They examined two isoforms of DVL, DVL-1 and DVL-3, which are localized to the nucleus to varying degrees in the breast cancer cell lines. Interestingly, they found that DVL-1 and DVL-3 occupy multiple active and inactive CYP19A1 promoters. Knockdown of DVL1 or DVL3 altered expression of different isoforms of CYP19A1 in both ER+ and ER- cell lines. Ultimately what is important for the biological effect of this transcriptional regulation is its effect on estrogen production. DVL3 knockdown trended to cause reduced levels of aromatase protein and significantly reduced estrogen levels and cellular proliferation. Altogether these results suggest that DVL isoforms differentially regulate expression of multiple tissue-specific CYP19A1 transcripts whose expression is dysregulated in breast cancers.

As a steroid hormone estrogen controls many normal aspects of human health such as glucose and lipid homeostasis, bone metabolism and brain function. Depletion of estrogen can lead to digestive issues, osteoporosis and cognitive problems. While aromatase inhibitors have been used successfully to treat breast cancer in postmenopausal women, they reduce aromatase activity in all tissues throughout the body causing side effects related to estrogen deficiency, including bone fracture, osteoporosis, and abnormal lipid metabolism [9]. Thus, understanding how tissue specific isoforms of CYP19A1 expression are regulated in normal cells and dysregulated in cancer cells is instrumental in refining treatment strategies to reduce unwanted side effects. The findings from this study provide the first evidence that DVL proteins participate in the regulation of transcription of tissue-specific aromatase transcripts [2]. Additional work is needed to further elucidate how signaling pathways altered in tumors can be targeted to reduce expression of CYP19A1 specifically in the tumor and its microenvironment while preserving expression in normal tissues.
